# Hospital Sunday Fund

**Published:** 1924-01

**Authors:** 


### HOSPITAL SUNDAY FUND.

THE Lord Mayor, as President and Treasurer of the Hospital Sunday Fund, took the chair at the annual meeting held at the Mansion House, on December 19. The Council's Report stated that the collection realised £93,892, which was £6,997 less than last year's total. Collections in places of worship totalled £43,167, £5,449 less than in 1922, and the donations £14,414, £1,041 less. The working expenses were £3,951, as against £4,335, the actual expenditure upon the collection and distribution being not greater than in pre-war days, in spite of the greatly increased cost of printing and of most of the essential elements in the fund's administration.

